# The c-Abl inhibitor, Radotinib HCl, is neuroprotective in a preclinical Parkinson’s disease mouse model

**DOI:** 10.1093/hmg/ddy143

**Published:** 2018-04-20

**Authors:** Saebom Lee, Sangjune Kim, Yong Joo Park, Seung Pil Yun, Seung-Hwan Kwon, Donghoon Kim, Dong Yeon Kim, Jae Soo Shin, Dae Jin Cho, Gong Yeal Lee, Hyun Soo Ju, Hyo Jung Yun, Jae Hong Park, Wonjoong Richard Kim, Eun Ah Jung, Seulki Lee, Han Seok Ko

**Affiliations:** 1 Neuroregeneration and Stem Cell Programs, Institute for Cell Engineering, Johns Hopkins University School of Medicine, Baltimore, MD 21205, USA; 2 Department of Neurology, Johns Hopkins University School of Medicine, Baltimore, MD 21205, USA; 3 The Russell H. Morgan Department of Radiology and Radiological Sciences, Johns Hopkins University School of Medicine, Baltimore, MD 21205, USA; 4 Adrienne Helis Malvin Medical Research Foundation, New Orleans, LA 70130, USA; 5 Central Research Institute, Il-Yang Pharmaceutical Co. Ltd., Yongin-si, Gyeonggi-do, Republic of Korea; 6 The Center for Nanomedicine at the Wilmer Eye Institute, Johns Hopkins University School of Medicine, Baltimore, MD 21205, USA; 7 Neuraly, Inc., Germantown, MD 20876, USA; 8 Diana Helis Henry Medical Research Foundation, New Orleans, LA 70130, USA

## Abstract

Accumulating evidence suggests that the non-receptor tyrosine kinase c-Abl plays an important role in the progression of Parkinson’s disease (PD) and c-Abl inhibition could be neuroprotective in PD and related α-synucleinopathies. Nilotinib, a c-Abl inhibitor, has shown improved motor and cognitive symptoms in PD patients. However, issues concerning blood–brain barrier (BBB) penetration, lack of selectivity and safety still remain. Radotinib HCl is a selective Bcr-Abl kinase inhibitor that not only effectively access the brain, but also exhibits greater pharmacokinetic properties and safety profiles compared to Nilotinib and other c-Abl inhibitors. Here, we show the neuroprotective efficacy of Radotinib HCl, a brain penetrant c-Abl inhibitor, in a pre-clinical model of PD. Importantly, *in vitro* studies demonstrate that the treatment of Radotinib HCl protects the α-synuclein preformed fibrils (PFF)-induced neuronal toxicity, reduces the α-synuclein PFF-induced Lewy bodies (LB)/Lewy neurites (LN)-like pathology and inhibits the α-synuclein PFF-induced c-Abl activation in primary cortical neurons. Furthermore, administration of Radotinib HCl inhibits c-Abl activation and prevents dopaminergic neuron loss, neuroinflammation and behavioral deficits following α-synuclein PFF-induced toxicity *in vivo*. Taken together, our findings indicate that Radotinib HCl has beneficial neuroprotective effects in PD and provides an evidence that selective and brain permeable c-Abl inhibitors can be potential therapeutic agents for the treatment of PD and related α-synucleinopathies.

## Introduction

Parkinson’s disease (PD) is the second most common, late onset, progressive neurodegenerative disorder and is characterized by selective loss of dopaminergic neurons in the substantia nigra pars compacta (SNpc), which causes motor dysfunction such as bradykinesia, rest tremor, and rigidity. To date, no proven neuroprotective therapy that can treat the disease or halt the progress of this disease is known (1). Although dopamine replacement alleviates the symptomatic motor dysfunction, its effectiveness is reduced as the disease progresses, leading to side effects such as severe motor fluctuations and dyskinesia. Moreover, this palliative therapeutic approach does not address the underlying mechanisms of the disease (2,3).

Oxidative stress is a prominent feature in sporadic PD, but the molecular mechanism linking oxidative stress to the progression of neuronal death and dysfunction remains unclear (4–6). Accumulating evidence suggests that the non-receptor tyrosine kinase, c-Abl is activated by oxidative stress and its activation plays a role in neurologic disorders including PD (7–9). Several lines of evidence have shown that c-Abl is activated in post-mortem PD brain tissue where there are indices of oxidative stress in areas such as the striatum and substantia nigra (10,11). Genetic depletion of c-Abl protects against loss of dopaminergic neurons in the MPTP (1-methyl-4-phenyl-1, 2, 3, 6-tetrahydropyridine) model of PD (12) and reduces α-synuclein aggregation, neuropathology, and neurobehavioral deficits in the A53T α-synuclein transgenic model of α-synucleinopathy (13). Recently, others and we have found that c-Abl phosphorylates α-synulcein at tyrosine 39 (Y39) enhancing α-synuclein aggregation and in part accumulation of Y39 α-synuclein is correlated with progression of PD (13,14). Thus, stress-induced activation of the non-receptor tyrosine kinase, c-Abl could contribute to the pathogenesis of sporadic PD and its inhibition could have neuroprotective effect in PD.

Pharmacological inhibition of c-Abl has shown beneficial neuroprotection in preclinical animal models of PD (15,16). Nilotinib, c-Abl inhibitor, reverses the loss of dopamine neurons and improves motor behavior in MPTP PD model (17) and α-synuclein-induced neurodegeneration mouse models (18). c-Abl inhibitors, Bafetinib (INNO-406) and Imatinib (Gleevec), have also shown beneficial effects in MPTP-induced loss of dopaminergic neurons and nigrostriatal toxicity (10,19). Importantly, although there are lack of a placebo control and a small cohort of PD patients, daily Nilotinib treatment has led to improved motor and cognitive symptoms in PD patients (20). While these evidences indicate that c-Abl inhibitors have potential for the chronic treatment of PD patients, many challenges including limited blood brain barrier (BBB) penetration and kinase selectivity remain in order to use c-Abl inhibitors as a disease-modifying drug for PD.

In this study, we tested therapeutic effect of Radotinib HCl, c-Abl inhibitor (Supect, Il-Yang Pharmaceutical Co., Ltd (IL-YANG PHARM.) of South Korea), which exhibits improved pharmacokinetic properties and BBB penetration compared to Nilotinib and other c-Abl inhibitors, in a preclinical α-synuclein preformed fibrils (PFF) model of sporadic PD. Here, we show that administration of Radotinib HCl inhibits c-Abl activation during disease progression, thereby ameliorating cardinal PD pathology in the α-synuclein PFF mouse model of sporadic PD.

## Results

### Treatment of Radotinib HCl reduces c-Abl activation induced by α-synuclein PFF *in vitro*

**Figure 1.**
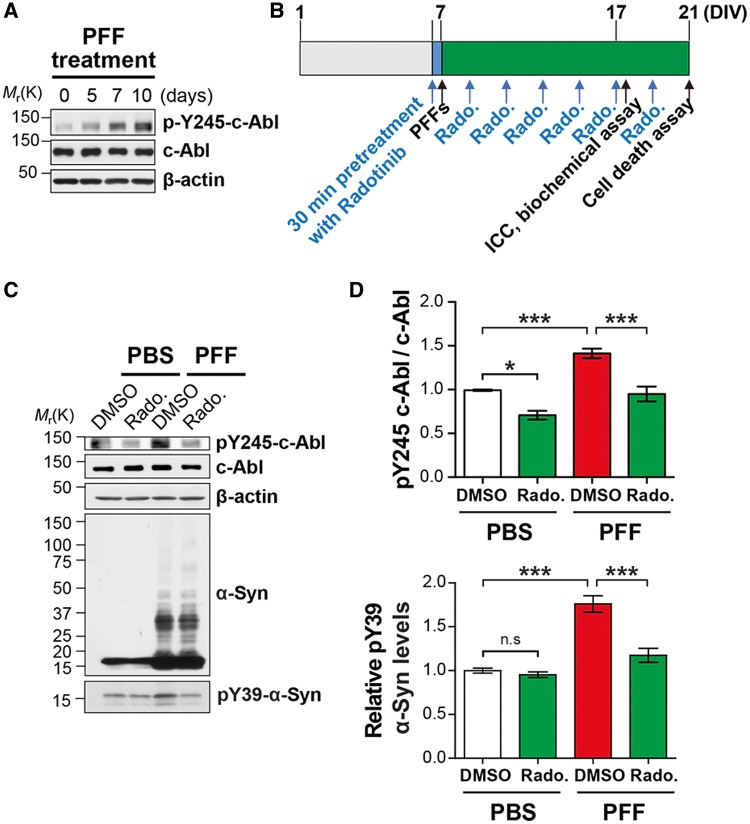
Radotinib HCl reduces α-synuclein PFF-induced c-Abl activation. (**A**) Western blot analysis of c-Abl activation by α-synuclein PFF treatment in cultured cortical neurons. (**B**) Experimental scheme. (**C**) Representative western blot data of reduced α-synuclein PFF-induced c-Abl activation by Radotinib HCl treatment (1 μM). (**D**) Quantification of pY245-c-Abl normalized to c-Abl. (**E**) Quantification pY39-α-synuclein normalized to β-actin. Bars indicate mean ± S.E.M. (*n* = 4). Statistical significance was determined by using one-way ANOVA with Tukey’s correction; *P < 0.05, ***P < 0.001. n.s., not significant. Radotinib, Radotinib HCl.

Accumulating evidence suggests that the non-receptor tyrosine kinase, c-Abl, is activated by oxidative stress and its activation is associated with neurodegenerative diseases including PD (21,22). Others and we showed the c-Abl is active in PD brain where there are indices of oxidative stress such as the striatum and substantia nigra (7–9). Moreover, recent studies suggest that Nilotinib, c-Abl inhibitor, reverses the loss of dopamine neurons and improves motor behavior in MPTP PD model (10,11). To firstly examine whether c-Abl contributes to the pathology of α-synuclein PFF mouse model of sporadic PD, primary cortical neurons were treated with α-synuclein PFF (1 μg/ml) or PBS and c-Abl activation was assessed using phospho-Tyr245 c-Abl (pY245-c-Abl) antibody. We observed that c-Abl is activated by treatment of α-synuclein PFF in a time-dependent manner (Fig. 1A). Next, to determine whether Radotinib HCl reduces c-Abl activation induced by α-synuclein PFF, lysates were prepared from α-synuclein PFF-treated neurons in the absence or presence of Radotinib HCl (1 μM) as depicted in Figure 1B and were utilized for Western blot analysis with pY245-c-Abl antibody. Notably, we observed that c-Abl activation induced by α-synuclein PFF was significantly decreased in the neurons with treatment of Radotinib HCl (1 μM) (Fig. 1C and D). c-Abl inhibition by Radotinib HCl was further assessed on the level of phosphotyrosine 39 (pY39)-α-synuclein, which shows increased propensity for aggregation and neuronal toxicity. The level of pY39-α-synuclein was increased in α-synuclein PFF-treated group while treatment of Radotinib HCl significantly decreased the increase in the levels of pY39-α-synuclein (Fig. 1C and E).

### Treatment of Radotinib HCl reduces α-synuclein PFF-induced LB/LN-like pathology *in vitro*

**Figure 2.**
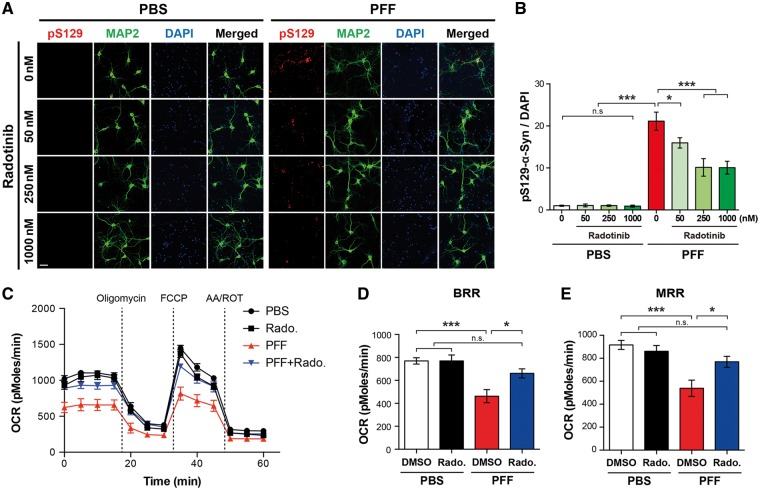
Radotinib HCl ameliorates α-synuclein PFF-induced α-synuclein aggregate formation and mitochondrial dysfunction. (**A**) Representative immunostaining images for p-α-synuclein (red) using pS129 antibody [MJF-R13 (8–8)] caused by α-synuclein PFF with or without Radotinib HCl (indicated concentrations) in primary cultured cortical neurons. The scale bar represents 50 μm. (**B**) Quantification of intensity of *p*-α-synuclein. Bars indicate mean ± S.E.M. (*n* = 3). (**C**) OCR was determined using the Seahorse XF24 Extracellular Flux Analyzer (Agilent Technologies, Santa Clara, CA). The basal respiratory rate (BRB) (**D**) and maximal respiratory rate (MRR) (**E**) were calculated (*n* = 8). Statistical significance was determined by using one-way ANOVA with Tukey’s correction; *P < 0.05, **P < 0.01, ***P < 0.001. n.s., not significant. Radotinib, Radotinib HCl.

A key feature of the α-synuclein PFF model is the pathologic α-synuclein aggregates in neurons (17). To determine whether Radotinib HCl reduces α-synuclein PFF-induced pathologic α-synuclein aggregates, Radotinib HCl was treated 30 min prior to α-synuclein PFF treatment with the indicated doses (three different concentrations: 50 nM, 250 nM and 1 μM). After 10 days, treated neurons were immunostained with pSer129 of a-synuclein antibody (MJF-R13 (8–8)). The Lewy bodies (LB)/Lewy neurites (LN)-like pathology induced by α-synuclein PFF was significantly decreased in neurons with treatment of Radotinib HCl in a dose-dependent manner (Fig. 2A and B). Together, these results indicate that Radotinib HCl reduces α-synuclein PFF-induced LB/LN-like pathology.

### Treatment of Radotinib HCl rescues α-synuclein PFF-induced mitochondrial dysfunction *in vitro*

Since α-synuclein PFF treatment induces mitochondrial dysfunctions in neurons (23,24), next we explored the role of Radotinib HCl in the mitochondrial abnormalities. DIV 7 primary cortical neurons were treated with α-synuclein PFF with or without Radotinib HCl (1 μM) and further incubated for 14 days. Serial injection of respiration modulators that target components of the electron transport chain (ETC) in the inner mitochondrial membrane help simplify cellular mitochondrial respiration as oxygen consumption rate (OCR). OCR was directly measured in primary cortical neurons treated with α-synuclein PFF in the presence or absence of Radotinib HCl (1 μM) using an XF24 Extracellular Flux Analyzer (Seahorse Bioscience) (Fig. 2C). We found that there were significant reductions in both basal respiration rate (BRR) (Fig. 2D) and maximal respiration rate (MRR) (Fig. 2E) in cortical neurons treated with α-synuclein PFF, which was markedly restored by treatment of Radotinib HCl. Taken together, these results indicate that Radotinib HCl rescues α-synuclein PFF-induced mitochondrial dysfunction.

### Treatment of Radotinib HCl protects against α-synuclein PFF-induced neuronal toxicity *in vitro*

**Figure 3.**
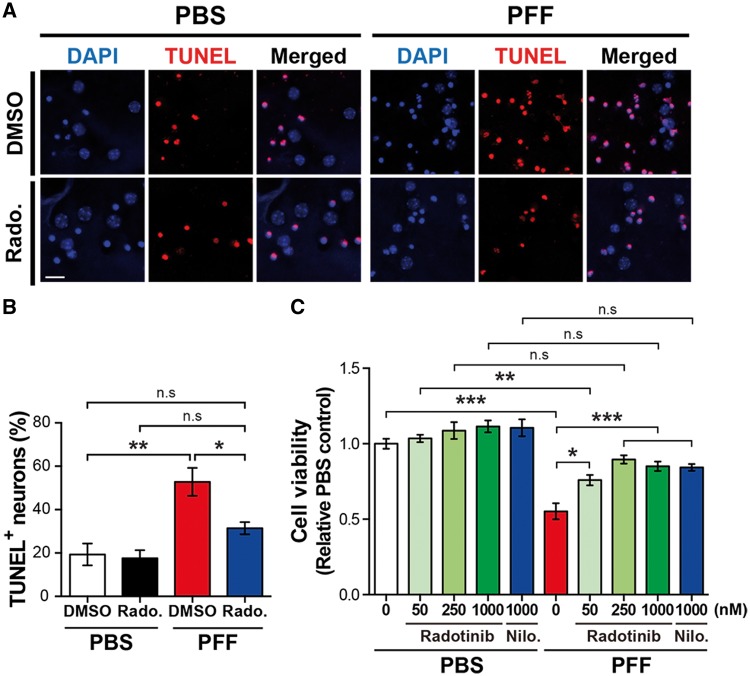
Radotinib HCl protects against α-synuclein PFF-induced neuronal toxicity. (**A**) Representative images of TUNEL staining in primary cortical neurons. Radotinib HCl was added at 1 μm. The scale bar represents 20 μm. (**B**) Quantification of TUNEL positive neurons. Bars indicate mean ± S.E.M. (*n* = 3–5). (**C**) The neuronal cell death was measured by Alamar Blue assay. Bars indicate mean ± S.E.M. (*n* = 4). Statistical significance was determined by using one-way ANOVA with Tukey’s correction and two-way ANOVA with Bonferroni’s multiple comparisons test; *P < 0.05, **P < 0.01, ***P < 0.001. n.s., not significant. Radotinib, Radotinib HCl. Nilo., Nilotinib.

Next, we investigated whether Radotinib HCl treatment rescues against neuronal death induced by α-synuclein PFF treatment. 7 DIV cortical neurons were treated with α-synuclein PFF for 14 days with and without Radotinib HCl with the respective doses, Nilotinib (1000 nM) as a positive control, and followed by multiple cell death assay to investigate active cell death (25,26). We observed that α-synuclein PFF treatment in neurons greatly increased the α-synuclein PFF toxicity compared to PBS-treated neurons, whereas Radotinib HCl treatment significantly attenuated the α-synuclein PFF neuronal toxicity in both TUNEL staining (Fig. 3A and B) and Alamar blue assay (Fig. 3C). Together, these data suggest that Radotinib HCl has a beneficial effect on PD neurotoxicity by preventing α-synuclein PFF-induced neuronal dysfunctions.

### Radotinib HCl ameliorates α-synuclein PFF-induced LB/LN-like pathology *in vivo*

Our *in vitro* studies strongly indicated that treatment of Radotinib HCl protects against the α-synuclein PFF-induced neuronal dysfunctions in primary neurons. Based on these exciting observations, pharmacological efficacy was validated in α-synuclein PFF mouse model of sporadic PD that best mimic PD in patients. In consistent with previous studies (27,28), α-synuclein PFF mouse model of sporadic PD recapitulates several key PD-like phenotypes including loss of dopamine neurons, dopaminergic motor deficits, and LB/LN-like pathology *in vivo*. Here, we assessed whether administration of Radotinib HCl in the mouse model could inhibit c-Abl activity during the disease progression and thereby reduce PD-like phenotypes *in vivo*.

**Figure 4.**
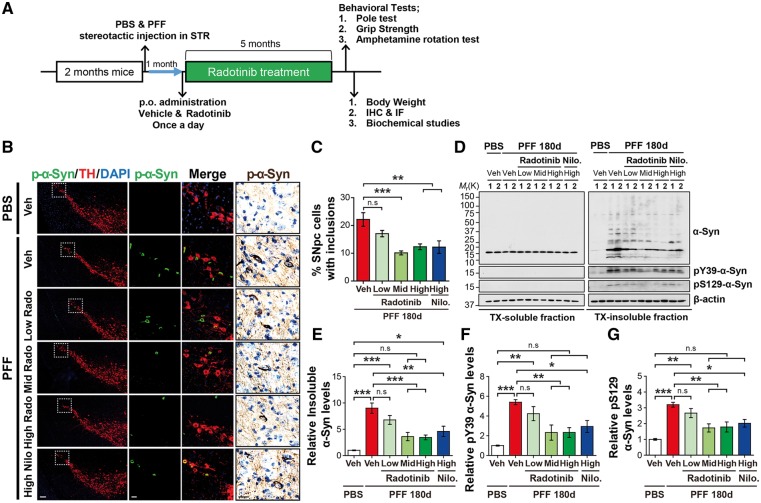
Radotinib HCl protects against α-synuclein PFF-induced pathology *in vivo*. (**A**) Preclinical study design of the α-synuclein PFF-induced PD mouse model. After 1 month, Radotinib HCl or Vehicle was delivered by oral administration at three different doses (3, 10 and 30 mg/kg) for 5 months. Nilotinib (30 mg/kg) was treated as a positive control. Mice were transcardially perfused after the last behavioral experiment. (**B**) Representative immunostaining for pS129-α-synuclein (green) and TH (red), immunohistological staining for pS129-α-synuclein (brown) in SNpc. The scale bars represent 100 μm (low magnification images) and 20 μm (high magnification images). (**C**) Quantification of SNpc regional neurons containing pS129-α-synuclein immunoreactive inclusions for each treatment group. Percentage of SNpc TH neurons with *p*-α-synuclein positive inclusion. Error bars represent the mean ± S.E.M, *n* = 4–6 mice per groups. (**D**) Representative immunoblots with sequential fractionation in 1% TX (TX-soluble) followed 2% SDS (TX-insoluble) in PBS and α-synuclein PFF stereotaxic injected mice treated with vehicle or Radotinib HCl and Nilotinib. (**E**) Quantification of levels of abnormal α-synuclein aggregates species, (**F**) pY39-α-synuclein and (**G**) pS129-α-synuclein in TX-insoluble fraction. Error bars represent the mean ± S.E.M, *n* = 4 mice per groups. One-way ANOVA was used to test for the statistical analysis, followed by Tukey’s correction; **P < 0.01, ***P < 0.001, n.s., not significant. Radotinib, Radotinib HCl. Nilo., Nilotinib.

To this end, α-synuclein PFF or PBS were injected unilaterally into the striatum of wild-type C57BL/6 at 2 months of age. One month after the intrastriatal injection of α-synuclein PFF (since spread and initial manifestation of α-synuclein pathology is beginning to be evident at the time point), these mice received Radotinib HCl treatment at three different doses (3 (low dose), 10 (mid dose) and 30 (high dose) mg/kg), once a day for 5 months by oral administration as depicted in Figure 4A. Nilotinib (30 (high dose) mg/kg) was also administered as a positive control. pS129-α-synuclein immunoreactivity was monitored in the SNpc with TH-positive neurons 180 days after α-synuclein PFF injection. We observed robust accumulation of pS129-α-synuclein in SNpc TH-positive neurons of α-synuclein PFF-injected mice at 180 days, which was significantly decreased in mice groups with Radotinib HCl at the mid and high doses and Nilotinib at the high dose as assessed by immunofluorescent analysis (Fig. 4B and C). Similar results were observed in the pS129-α-synuclein immunoreactivity as assessed by immunohistological staining (Fig. 4B).

To further explore the levels of pS129-α-synuclein and pY39-α-synuclein, ventral midbrain tissues were fractionated into Triton X-100 (TX) soluble and insoluble fractions. α-Synuclein levels were unchanged through all groups in the TX-soluble fraction, but accumulation of abnormal α-synuclein aggregates species and increase in the levels of pS129-α-synuclein and pY39-α-synuclein were observed in the TX-insoluble fraction of α-synuclein PFF-injected mice (Fig. 4D–G), which was significantly reduced in α-synuclein PFF-injected mice treated with Radotinib HCl in a dose-dependent manner. Interestingly, at high dose, Nilotinib showed less effect on reduction in the accumulation of the TX-insoluble α-synuclein aggregate species and the increase in the levels of pS129-α-synuclein and pY39-α-synuclein compared to Radotinib HCl (Fig. 4D–G). These results taken together indicate that Radotinib HCl reduces the LB/LN-like pathology induced by α-synuclein PFF *in vivo*.

### Radotinib HCl prevents against α-synuclein PFF-induced dopaminergic neurodegeneration and dopaminergic terminal loss *in vivo*

**Figure 5.**
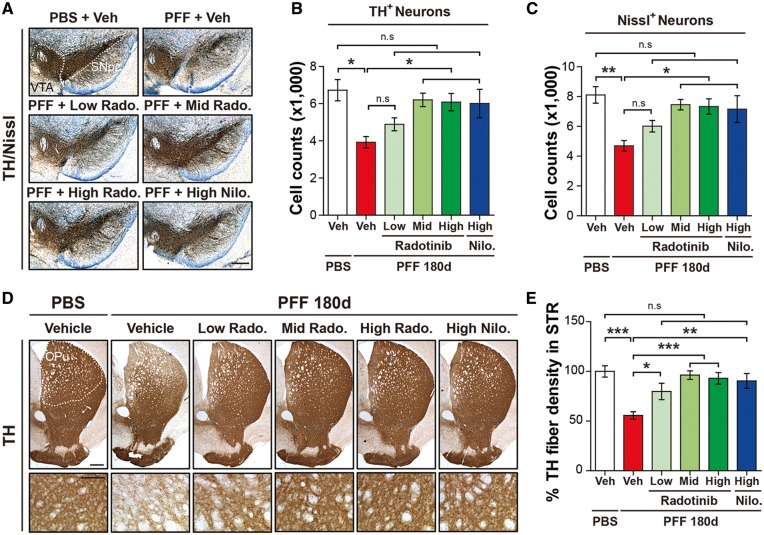
Radotinib HCl protects against α-synuclein PFF-induced dopaminergic neurodegeneration and fiber density. (**A**) Representative photomicrographs from coronal mesencephalon sections containing TH-positive neurons in PBS and α-synuclein PFF stereotaxic injected mice treated with vehicle or Radotinib HCl and Nilotinib. The scale bar represents 500 μm. (**B**) Stereology counts of TH and (**C**) Nissl-positive neurons in the SNpc region. Unbiased sterologic counting was performed in SNpc region. Error bars represent the mean ± S.E.M, *n* = 4–6 mice per groups. (**D**) Representative photomicrograph of striatal sections stained for TH immunoreactivity. High power view of TH fiber density in the striatum (lower panels). The scale bars represent 100 μm (upper panles) and 50 μm (lower panels). CPu and STR, Striatum. (**E**) Quantification of dopaminergic nerve fiber densities in the striatum by using Image J software (NIH). Error bars represent the mean ± S.E.M, *n* = 4–6 mice per groups. One-way ANOVA was used to test for the statistical analysis, followed by Tukey’s correction; *P < 0.05, **P < 0.01, ***P < 0.001, n.s., not significant. Radotinib, Radotinib HCl. Nilo., Nilotinib.

To examine the efficacy of Radotinib HCl in nigrostriatal degeneration in the α-synuclein PFF mouse model, we identified TH-positive neurons in the SNpc and their fibers in the striatum by immunohistochemistry. Unbiased stereologic counting of SNpc TH-and Nissl-positive neurons revealed significant loss of dopaminergic neurons in mice at 180 days after α-synuclein PFF post-injection (Fig. 5A–C). In contrast, there was a dramatic preservation of dopaminergic neurons in SNpc TH- and Nissl-positive neurons of mice group following Radotinib HCl treatment at the mid and high doses and Nilotinib at the high dose (Fig. 5A–C). Striatal fiber density was examined via optical densitometry using Image J software (NIH). α-Synuclein PFF treatment significantly reduced dopaminergic nerve terminal integrity compared to PBS-treated controls (Fig. 5D and E). In contrast, administration of Radotinib HCl at the low, mid and high doses significantly rescued the α-synuclein PFF-induced reduction in TH-fiber density (Fig. 5D and E). Similar results were observed in mice with Nilotinib at the high dose.

### Radotinib HCl reduces α-synuclein PFF-induced neuroinflammation *in vivo*

**Figure 6.**
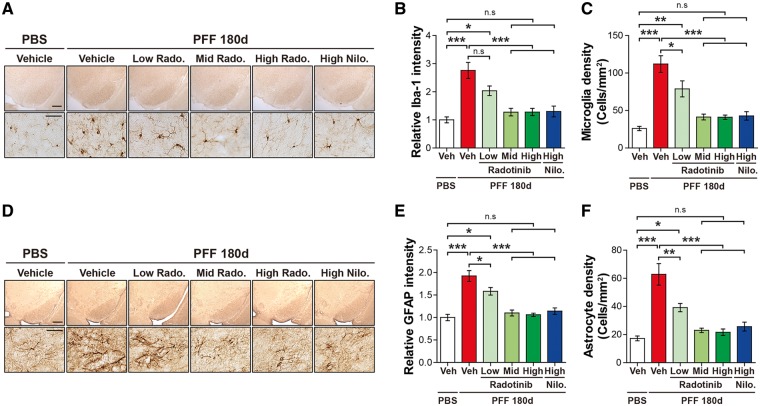
Radotinib HCl restores α-synuclein PFF-induced neuroinflammation. (**A**) Representative images of immunohistochemistry data for Iba-1 in PBS and α-synuclein PFF stereotaxic injected mice treated with vehicle or Radotinib HCl and Nilotinib. The scale bars represent 500 μm (upper panels) and 50 μm (lower panels). (**B**) The intensity of Iba-1 positive signals in the SNpc and (**C**) quantification of Iba-1 positive cell number in SNpc. The intensity of Iba-1 immunoreactivity and densities of microglia in the SNpc region were measured with ImageJ software. Error bars represent the mean ± S.E.M, *n* = 4–6 mice per groups. (**D**) Representative images of immunohistochemistry data for GFAP in PBS and α-synuclein PFF stereotaxic injected mice treated with vehicle or Radotinib HCl and Nilotinib. The scale bars represent 500 μm (upper panles) and 50 μm (lower panels). (**E**) The intensity of GFAP positive signals in the SNpc and (**F**) quantification of GFAP positive cell number in SNpc. The intensity of GFAP immunoreactivity and densities of astrocyte in the SNpc region were measured with ImageJ software. Error bars represent the mean ± S.E.M, *n* = 4–6 mice per groups. One-way ANOVA was used to test for the statistical analysis, followed by by Tukey’s correction; *P < 0.05, **P < 0.01, ***P < 0.001, n.s., not significant. Radotinib, Radotinib HCl. Nilo., Nilotinib.

To determine whether α-synuclein PFF induces microglia activation *in vivo*, ionized calcium-binding adapter molecule 1 (Iba-1) immunoreactivity was measured. Intrastriatal injection of α-synuclein PFF significantly increased Iba-1 immunoreactivity and the number of activated microglia with rounded cell bodies and short, thickened processes in the SNpc 180 days after α-synuclein PFF post-injection (Fig. 6A–C). In contrast, Radotinib HCl at the mid and high doses and Nilotinib at the high dose significantly reduced the Iba-1 immunoreactivity and microglial density (Fig. 6A–C). To assess the effect of Radotinib HCl on α-synuclein PFF-induced astrocyte activation, glial fibrillary acidic protein (GFAP) staining was conducted within the SNpc regions of all groups. Intrastriatal injection of α-synuclein PFF significantly increased GFAP immunoreactivity and enhanced astrocyte activation with increased soma size in the SNpc 180 days after α-synuclein PFF post-injection (Fig. 6D–F). The astrocyte activation induced by α-synuclein PFF was significantly decreased in groups treated with Radotinib HCl at the mid and high doses and Nilotinib at the high dose (Fig. 6D–F). Taken together, these data suggest that α-synuclein PFF-induced neuroinflammation is restored by Radotinib HCl in the SNpc region.

### Radotinib HCl rescues α-synuclein PFF-induced behavioral deficits *in vivo*

**Figure 7.**
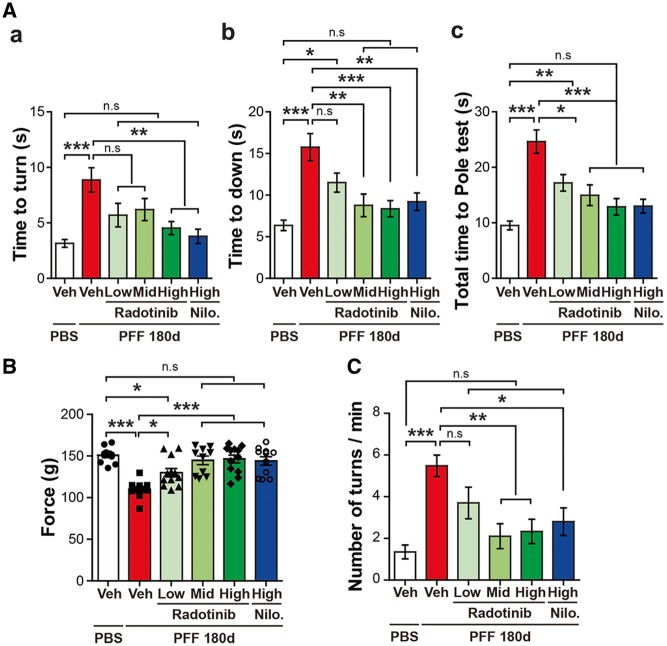
Radotinib HCl protects against α-synuclein PFF-induced behavioral deficits (**A–C**) 6 months after the α-synuclein PFF or PBS stereotaxic brain injection, the behavioral tests were performed in Vehicle and Radotinib HCl treated mice. Nilotinib-treated group as a positive control. Behavioral abnormalities were improved in mice administered with Radotinib HCl. Results of animals on the (A) Pole test, (B) grip strength, (C) amphetamine rotation test. Error bars represent the mean ± S.E.M (*n* = 9–12 mice per each group). One-way ANOVA was used for statistical analysis followed by Tukey’s correction; *P < 0.05, **P < 0.01, ***P < 0.001, n.s., not significant. Radotinib, Radotinib HCl. Nilo., Nilotinib.

To determine whether administration of Radotinib HCl prevents against α-synuclein PFF-induced dopaminergic motor deficits, behavioral assays including the pole test, the grip strength, and the amphetamine rotation test were performed at 6 months after the α-synuclein PFF post-injection. For the pole test, time to turn down and total time taken to reach the base of the pole were analyzed. The latency induced by α-synuclein PFF was significantly restored by Radotinib HCl at the low, mid and high doses (Fig. 7A). For the grip strength test, the maximum fore-limb holding strength was recorded by the force transducer. Neuromuscular strength was reduced by α-synuclein PFF injection, which was restored by Radotinib HCl at the low, mid and high doses (Fig. 7B). Assessment of drug-induced rotational behavior was monitored following amphetamine administration (5 mg/kg, I.P.). Mice were placed in individual white bowls with a diameter of 20 cm and allowed to habituate to their environment for 10 min before turns contralateral and ipsilateral to the lesion were recorded over 20 min. Increase in the number of full body ipsilateral rotations was observed following α-synuclein PFF injection, which was reduced by Radotinib HCl at the mid and high doses (Fig. 7C). Intriguingly, Nilotinib at the high dose also protected against the behavioral deficits in the α-synuclein PFF mouse model of sporadic PD. Taken together, our findings suggest that Radotinib HCl prevents α-synuclein PFF-induced LB/LN like pathology, dopaminergic neurodegenerations, and neuroinflammation, as well as having neuroprotective effects against α-synuclein PFF-induced motor behavioral deficits *in vivo*.

## Discussion

c-Abl activation could contribute to the neurodegeneration in PD through several different pathways including tyrosine phosphorylation of parkin leading to impaired parkin function and tyrosine phosphorylation of α-synuclein leading to enhanced α-synuclein aggregation (11,13). Thus, c-Abl inhibitors are very attractive disease-modifying agents for the treatment of PD and related α-synucleinopathies. In this study, we confirm the therapeutic effect of brain-penetrant c-Abl inhibitor, Radotinib HCl, in α-synuclein PFF neuronal model as well as in mouse model of sporadic PD and extend on prior studies that inhibition of c-Abl could protect to progress PD pathologies. Our findings indicate that c-Abl inhibition with Radotinib HCl *in vitro* restores impaired mitochondrial respiration and decreases the formation of pathologic α-synuclein aggregates induced by α-synuclein PFF. Also, c-Abl inhibition with Radotinib HCl *in vivo* protects against α-synuclein PFF-induced loss of dopaminergic neurons reduction in striatal dopaminergic nerve terminal density and neuroinflammation and rescues behavior deficits in a dose-dependent manner. The degree of protection by Radotinib HCl against α-synuclein PFF-induced neurodegeneration seems to be greater than the c-Abl inhibitors, Imatinib and Nilotinib, which is probably due to effective brain penetration of Radotinib HCl. These observations suggest that Radotinib HCl could be possibly adapted as a therapy for PD.

There are a number of c-Abl inhibitors for treatment of chronic myeloid leukemia (CML) (29). Among them, Imatinib, Nilotinib and Bafetinib have been validated in pre-clinical models of PD as a disease-modifying agent. However, selectivity, limited BBB penetration, and toxicity remain to be concerns with these inhibitors. Radotinib HCl used in the current study is a second-generation Bcr-Abl tyrosine kinase inhibitor (TKI), resembling structure with Imatinib and close to Nilotinib (30). Compared to other multitarget TKIs such as Dasatinib (BMS-354825, Bristol-Myers Squibb) (31) and Bosutinib (SKI-606, Pfizer) (32), Radotinib HCl and Nilotinib selectively inhibit BCR-Abl with IC_50_ of 34 nM (33) and less than 30 nM (34), respectively. Unlike Imatinib, Nilotinib is more potent with moderate brain penetration attracting as a potential treatment for neurological disorders (35,36). Consistent to structural similarity between Nilotinib and Radotinib HCl (30), we observed that Radotinib HCl is detected 3.3 times higher than Nilotinib in brain tissue after single oral administration suggesting that Radotinib HCl possesses more effective brain-penetrating property (Table 1). Consistent with this notion that α-synuclein PFF-induced c-Abl activation (Supplementary Material, Fig. S1A and B), accumulation of the TX-insoluble α-synuclein aggregates species (Fig. 4D and E), and phosphorylation of c-Abl substrates including Y39-α-synuclein (Fig. 4D and F) and p38 MAPK (Supplementary Material, Fig. S1A and C) were substantially decreased in mice treated with Radotinib HCl compared to those in mice treated with Nilotinib. In the current study, we demonstrate that Radotinib HCl recovers reduction in dopaminergic nerve terminal integrity, and rescues behavioral deficits in the post test (Fig. 7) at the low dose (3 mg/kg *in vivo*) indicating a dose (human equivalent dose = 14.6 mg/60 kg). These observations suggest that Radotinib HCl can be administrated at a lower dose and possibly for prolonged period with fewer risks and side effects.

**Table 1.** Pharmacokinetic parameters of Radotinib HCl and Nilotinib in plasma and brain tissue

Animal no.	Radotinib HCl				Nilotinib			
	Plasma		Brain tissue		Plasma		Brain tissue	
	AUC_t_ (ng·h/ml)	C_max_ (ng/ml)	AUC_t_ (ng·h/ml)	C_max_ (ng/ml)	AUC_t_ (ng·h/ml)	C_max_ (ng/ml)	AUC_t_ (ng·h/ml)	C_max_ (ng/ml)
**1**	62 294.7	9 680.1	591.3	70.7	87 985.3	18 157.3	164.0	34.4
**2**	74 356.2	11 135.4	576.5	101.5	77 472.1	16 878.9	155.7	35.5
**3**	124 593.5	9 433.2	772.4	61.5	175 949.6	24 529.6	272.5	38.0
**Mean**	87 081.4	10 082.9	646.7	77.9	11 3802.3	19 855.2	197.4	35.9
**SD**	33 041.4	919.8	109.1	21.0	54 077.2	4 098.3	65.1	1.9

It is plausible that Radotinib HCl protection against α-synuclein PFF-induced neurodegeneration occurs through a reduction in tyrosine phosphorylation of parkin, leading to accumulation of parkin substrates (11) and a reduction in tyrosine phosphorylation of α-synuclein leading to enhanced α-synuclein aggregation (13,14) as well as inhibition of neuroinflammation (13,37) and functional recovery of damaged mitochondria (23,24,38). Consistent with this notion is the observation that treatment of Radotinib HCl leads to recovery in impaired mitochondrial respiration and decreases in the formation of pathologic α-synuclein aggregates and the levels of pY39-α-synuclein induced by α-synuclein PFF as well as reduction in neuroinflammation *in vivo*. Recent studies have reported that c-Abl activation contributes to lipopolysaccharide-primed rotenone-induced microglial activation through the activation of the NLRP3 (NACHT, LRR and PYD domains containing protein 3) inflammasome signaling (37). Other report further suggests that c-Abl activation is associated with increase in the levels of phosphorylated p38 MAPK, a substrate for c-Abl in microglia of MPTP-treated mice (12). In the current study, we found that the increase in phosphorylation of-c-Abl (Y245) was accompanied by an increase in phosphorylation of p38 MAPK in α-synuclein PFF-injected mice, which was reversed by Radotinib HCl treatment (Supplementary Material, Fig. S1). Further studies will be required to test the possibility that c-Abl activation-mediated signaling cascade may play an important role in neuroinflammatory processes and thereby contributing to PD pathogenesis.

Our current findings coupled with other recent observations provide that pharmacological inhibition of c-Abl could be an attractive disease-modifying agent for PD treatment (17,20). In recent clinical trial, moderately advanced PD patients who were treated with Nilotinib for 6 months showed improved motor and cognitive symptoms although the main purpose of this study was to check for the safety of using this chemotherapy medication (20) (Clinicals.gov NCT02281474). A Phase II placebo-controlled study on Nilotinib in PD is ongoing (recruiting). Although only proven in a small number of patients, Nilotinib demonstrates potential as a first in class disease-modifying drug in PD. Thus, other c-Abl inhibitors with better pharmacokinetic properties and safety profiles may need to be identified to take advantage of inhibition of c-Abl as a disease-modifying therapy for PD. Our study provides therapeutic effect and pharmacokinetic properties of brain-penetrant Radotinib HCl, as well as additional clues for the inhibition of c-Abl that play a role in α-synuclein PFF model of sporadic PD. Since c-Abl activation is linked to many neurodegenerative disorders including Alzheimer’s disease (39) and amyotrophic lateral sclerosis (40), Radotinib HCl may also have broad clinical implications. Therefore, the next step will be to conduct clinical trial to assess Radotinib HCl efficacy in PD patients and related α-synucleinopathies.

## Materials and Methods

### Animals

C57BL/6 mice were obtained from Jackson Laboratory (ME, USA). All procedures involving animals were approved by and conformed to the guidelines of the Institutional Animal Care Committee of Johns Hopkins University (Protocol Number: M017M100). Animals were housed in a 12 h dark and light cycle with free access to water and food. All mice were acclimatized in the procedure room before starting any animal experiments. We have taken great measures to reduce the number of animals used in these studies and also taken effort to reduce animal suffering from pain and discomfort.

### α-Synuclein purification and α-synuclein PFF preparation

Recombinant α-synuclein proteins were purified as previously described (28,41). α-Synuclein PFF was prepared by agitating α-synuclein in a transparent glass vial with a magnetic stirrer (1000 rpm at 37°C). After 7 days of incubation, the α-synuclein aggregates were sonicated for 30 s at 10% amplitude (Branson Digital Sonifier, Danbury, CT). α-Synuclein PFF was kept at −80°C until assayed.

### Drug preparation

Radotinib HCl (IL-YANG PHARM.) and Nilotinib (APEX Bio) were dissolved in dimethyl sulfoxide (DMSO, Sigma-Aldrich) for the cell treatment. Carboxymethylcellulose sodium salt (0.5%) solution (Sigma-Aldrich) was used as vehicle for oral administration.

### Blood brain barrier penetration of Radotinib HCl and Nilotinib in mice

This study was performed in the Department of Pharmacology and Toxicology, IL-YANG PHARM. Radotinib HCl and Nilotinib were suspended in 0.5% carboxymethyl cellulose at 50 mg/kg/10 ml, and were administered up to 6 weeks, female, ICR mice (Orient Bio. Gapyeong, Gyeonggi-do, Korea) by oral gavage. At 1, 2, 3, 4, 6, 8 and 24 h post dose, blood samples were collected by venipuncture of abdominal vena cava under anesthesia with Zoletil 50 (Virbac)/Rompun (Bayer). All blood samples were delivered into heparin tube (Fujifilm) and plasma was obtained by centrifugation at 3000 rpm, 15°C for 10 min. After blood sample collection, mice were perfused transcardially with 80 ml of PBS and whole brain was isolated. Whole brain was weighed and homogenized in PBS (1: 3 dilution). Then, 2-folds of acetonitrile were added to brain tissue sample and the mixture was centrifuged at 14 000 rpm, 4°C for 5 min. Supernatant was used for analysis. The concentration of Radotinib HCl and Nilotinib in plasma and brain tissue were measured using LC-MS/MS.

### Primary neuron cultures and α-synuclein PFF and Radotinib HCl treatment

C57BL/6 mice were obtained from the Charles River (Wilmington, MA). Primary cortical neurons were prepared from E15.5 pups and cultured in Neurobasal media supplemented with B-27, 0.5 mM l-glutamine, penicillin and streptomycin (Invitrogen, Grand Island, NY) on 24-well tissue culture plates coated with poly-d-lysine. Radotinib HCl was added to neurons for 30 min and α-synuclein PFF (final concentration 1 μg/ml) was further incubated as described (10 or 14 days) followed by Immunocytochemistry and biochemical or toxicity assays. Radotinib HCl was added to neurons three times a week before the assays.

### Western blot analysis

After washing two times with PBS, primary cortical neurons were harvested and then lysed with RIPA lysis buffer (Thermo Scientific) with phosphatase inhibitor cocktail (Sigma-Aldrich) and complete protease inhibitor mixture (Roche) for 30 min in ice. The lysate is then homogenized and centrifuged at 22 000 × *g* for 30 min.

The mouse brain tissues were homogenized and prepared in lysis buffer (10 mM Tris–HCL, pH 7.4, 150 mM NaCl, 5 mM EDTA, 0.5% Nonidet P-40, 10 mM Na-β-glycerophosphate, phosphatase inhibitor cocktail (Sigma-Aldrich) and complete protease inhibitor mixture (Roche)), using a Diax 900 homogenizer (Sigma-Aldrich). After homogenization, samples were rotated at 4°C for 30 min for complete lysis, the homogenate was centrifuged at 22 000 × *g* for 30 min and the supernatants were collected.

For Triton X-100 (TX, Sigma-Aldrich) soluble and insoluble fraction, cells were prepared with sequential lysis buffer. Samples were homogenized in the followed TX-soluble buffer (50 mM Tris (pH 8.0), 150 mM NaCl, 1% TX with phosphatase inhibitor cocktail and protease inhibitor cocktail) and then were centrifuged and collected the soluble supernatant. The insoluble pellet was resuspended in TX-insoluble buffer (50 mM Tris (pH 8.0), 150 mM NaCl, 1% TX, 2% SDS with phosphatase inhibitor cocktail and protease inhibitor cocktail) and then was sonicated and centrifuged at 22 000 × *g* for 30 min.

Protein concentrations were determined using the BCA assay (Pierce, Rockford, IL, USA). 2X Laemmli buffer (Bio-Rad) was utilized to dilute the lysates. Equal amounts of lysates were separated on 8–16% gradient SDS-PAGE gels (Life technologies) and transferred to nitrocellulose membrane. Membrane was blocked with TTBS (150 mM NaCl, 10 mM Tris–HCl (pH 7.4), 0.05% Tween 20) containing 5% skim milk for 30–60 min and incubated at 4°C overnight with primary antibodies; mouse anti-α-synuclein (610787; BD Transduction Laboratories), rabbit anti-pY245 c-Abl (2861; Cell Signaling Technology), mouse anti-c-Abl (554148; BD Transduction Laboratories), rabbit monoclonal anti-pS129 α-synuclein (ab168381; Abcam), rabbit polyclonal anti-phospho-p38 MAPK (Thr180/Tyr182) (9211; Cell Signaling Technology), rabbit polyclonal anti-p38 MAPK (A11340; Abclonal Technology) followed by HRP-conjugated rabbit or mouse secondary antibodies (1: 5000, GE Healthcare) for 1 h at room temperature. Rabbit polyclonal anti-pY39-α-synuclein antibody was generated as described previously (13). Protein bands were detected using chemiluminescence detection system onto X-ray film. The membranes were reprobed with HRP-conjugated β-actin antibody (1: 20 000, Sigma-Aldrich).

### Immunofluorescent analysis

Primary mouse cortical neurons were plated onto poly-d-lysine coated coverslips. 4% paraformaldehyde was used to fix the neurons, followed by blocking in a solution with 5% normal donkey serum (Jackson ImmunoResearch), 2% BSA (Sigma-Aldrich) and 0.1% TX (Sigma-Aldrich) for 30–60 min at room temperature. Floating brain tissue sections were blocked with 10% donkey serum (Jackson Immunoreserach) in PBS plus 0.2% TX for 30 min at room temperature.

Primary antibodies, anti-pS129-α-synuclein (ab168381; Abcam), anti-TH antibody (Novus Biologicals) and anti-MAP2 (MAB3418; Millipore) were incubated at 4°C overnight. The samples were washed with PBS three times, followed by 1 h of incubation with a mixture of FITC-conjugated (Jackson ImmunoReserach) and Cy3-conjugated (Jackson ImmunoReserach) secondary antibodies at room temperature. The fluorescent images were acquired via a Zeiss confocal microscope (Zeiss Confocal LSM 710) after the coverslips were mounted on microscope slides.

### Determination of oxygen consumption rate

OCR was determined using the Seahorse XF24 Extracellular Flux Analyzer and the XF Cell Mito Stress Test Kit according to the manufacturer’s instruction (Agilent Technologies, Santa Clara, CA) (42). Briefly, primary mouse cortical neurons were plated in a XF24 cell culture microplate coated with poly-d-lysine. Cells were washed once with XF Assay Media, pre-incubated in a non-CO_2_ incubator at 37°C for 30 min in XF Assay Media. For the assay, cells were treated sequentially with 1 μM Oligomycin, 2 μM carbonyl cyanide-*p*-trifluoromethoxyphenylhydrazone (FCCP), and 1 μM Rotenone plus 1 μM Antimycin A. Oligomycin inhibits ATP synthase (complex V) to measure ATP production and proton leak. FCCP uncouples oxygen consumption from ATP production enables to measure maximal respiration. Rotenone and antimycin A inhibit complexes I and III and shut down mitochondrial respiration. Basal respiration was calculated using the mean of the OCR measurements before oligomycin injection minus the mean of the OCR measurements after rotenone and antimycin A injection, and maximal respiration was defined as the difference between the mean of the OCR measurements after FCCP injection and the mean of the OCR measurements after rotenone and antimycin A injection.

### Cell viability analysis

Cell viability was tested via two methods; In Situ Cell Death Detection Kit (Roche) for TUNEL assay and Alamar Blue assay (Thermo Fisher). Primary cultured cortical neurons were treated with α-synuclein PFF with or without Radotinib HCl (0, 50, 250, 1000 nM) or Nilotinib (1000 nM) for 14 days. Cell death was assessed through the TUNEL assay, according to the manufacturer’s protocol. For Alamar Blue assay, after adding Alamar Blue solution for 4 h, cell viability was determined by fluorescence at excitation wavelength 570 nm and emission wavelength 585 nm.

### Stereotaxic α-synuclein PFF injection

For stereotaxic injection of α-synuclein PFF, 2 months old mice were anesthetized with the mixture of xylazene and ketamine. An injection cannula (26.5 gauge) was applied stereotaxically into the striatum (anteroposterior, 2.0 mm from bregma; mediolateral, 0.2 mm; dorsoventral, 2.6 mm) unilaterally into the right hemisphere. The infusion of 2 μl α-synuclein PFF (2.5 μg/μl in PBS) or PBS was performed at a rate of 0.4 μl per min. The head skin was closed by suturing and wound healing and recovery were monitored following surgery. For stereological analysis, animals were perfused and fixed intracardially with ice-cold PBS followed by 4% paraformaldehyde at 6 months after striatal α-synuclein PFF injections. The brain was removed and processed for immunohistochemistry or immunofluorescence. Behavioral test was performed at 6 months after the unilateral striatal α-synuclein PFF injections. Treatment of Radotinib HCl was accomplished after 1 month of striatal α-synuclein PFF injections, once a day.

### Behavioral test

#### Pole test

Before the actual test, mice were trained for three consecutive days and each training session consists of three test trials. Briefly, the mice were placed on the top of the pole (3 in. from the top of the pole) and then total time to reach the bottom of the pole was recorded. For the test, mice went through three sessions and the total time was recorded. Results were recorded by turn down, climb down, and total time (in seconds).

#### Grip strength

Neuromuscular strength was measured by maximum holding force generated by the mice. Mice were allowed to grasp a metal grid with their forelimbs. The tail was gently pulled and the maximum holding force recorded by the force transducer (Biosed, USA) when the mice released their grasp on the grid. The peak holding strength was digitally recorded and displayed as force in grams.

#### Amphetamine-induced stereotypic rotation test

The amphetamine treatment (5 mg/kg, Sigma-Aldrich) was intraperitoneally administered after 6 months of α-synuclein PFF injections with or without Radotinib HCl treatment. Mice were placed in individual white bowls with a diameter of 20 cm and allowed to habituate to their environment for 10 min before turns contralateral and ipsilateral to the lesion were recorded over 20 min. Full-body ipsilateral rotations (clockwise) per 1 min session were recorded for each mouse from the video recordings.

### Immunohistochemistry and quantitative analysis

The perfusion was performed with ice-cold PBS and fixed by 4% paraformaldehyde after deep anesthesia. Whole brains were collected, and fixed in the 4% paraformaldehyde, and incubated in cryoprotection solution (30% sucrose/PBS, pH 7.4). Brains were frozen in an optimal cutting temperature (OCT) solution and serial coronal sections (30 µm sections) were cut with a microtome. Free-floating sections were blocked with 5% goat or horse serum/PBS plus 0.2% Triton X-100 and incubated with anti-TH antibody (Novus Biologicals) or anti-pS129-α-synuclein followed by incubation with biotin-conjugated anti-rabbit antibody or anti-mouse antibody (Vector Labs). After washing three times, ABC reagents (Vector Labs) were added and sections were developed using SigmaFast 3, 3′-Diaminobenzidine (DAB) Peroxidase Substrate (Sigma-Aldrich). Sections were counterstained with Nissl (0.09% thionin). TH-positive and Nissl-positive dopaminergic neurons from the SNpc region were counted through optical fractionators, the unbiased method for cell counting. This method was carried out by using a computer-assisted image analysis system consisting of an Axiophot photomicroscope (Carl Zeiss Vision) equipped with a computer-controlled motorized stage (Ludl Electronics), a Hitachi HV C20 camera and Stereo Investigator software (MicroBright-Field). The total number of TH-stained neurons and Nissl counts was analyzed as previously described (27,43). Fiber density in the striatum was analyzed by optical density (OD) measurement. ImageJ software (NIH) was used to analyze the OD as previously described (44). The number of pS129-α-synuclein positive and Nissl positive in the SNpc region were measured with ImageJ software and field counting. Microglia and astrocyte from the SNpc region were stained with anti-Iba-1 (Wako) for microglia or anti-GFAP (Dako) antibodies for astrocyte followed by incubation with biotin-conjugated anti-rabbit antibody and ABC reagents. And then sections were developed using SigmaFast DAB Peroxidase Substrate (Sigma-Aldrich). The number and immunoreactivities of microglia and astrocyte in the SNpc region were measured with ImageJ software.

### Statistical analysis

All data were analyzed using GraphPad Prism 6 software. Differences between two means were assessed by a paired or unpaired *t*-test. Differences among multiple means were assessed, as indicated, by one-way ANOVA or two-way ANOVA followed by Tukey’s post-hoc test or by Bonferroni’s multiple comparisons test as appropriate. Error bars represent S.E.M., as indicated. Probabilities of *P* < 0.05 or as indicated were considered statistically significant.

## Supplementary Material

Supplementary Material is available at *HMG* online.

## Supplementary Material

Supplementary Data
