# Supplementary material for: The c-Abl inhibitor, Radotinib HCl, is neuroprotective in a preclinical Parkinson’s disease mouse model
Source: Hum Mol Genet. 2018 Apr 20;27(13):2344–56. doi: 10.1093/hmg/ddy143 (PMC6005030; doi:10.1093/hmg/ddy143)
Supplement: Supplementary Data [file ddy143_supplementary_material_lee_et_al_hmg_(hmg-2018-twb-0055r).pdf]

Supplementary Figure S1

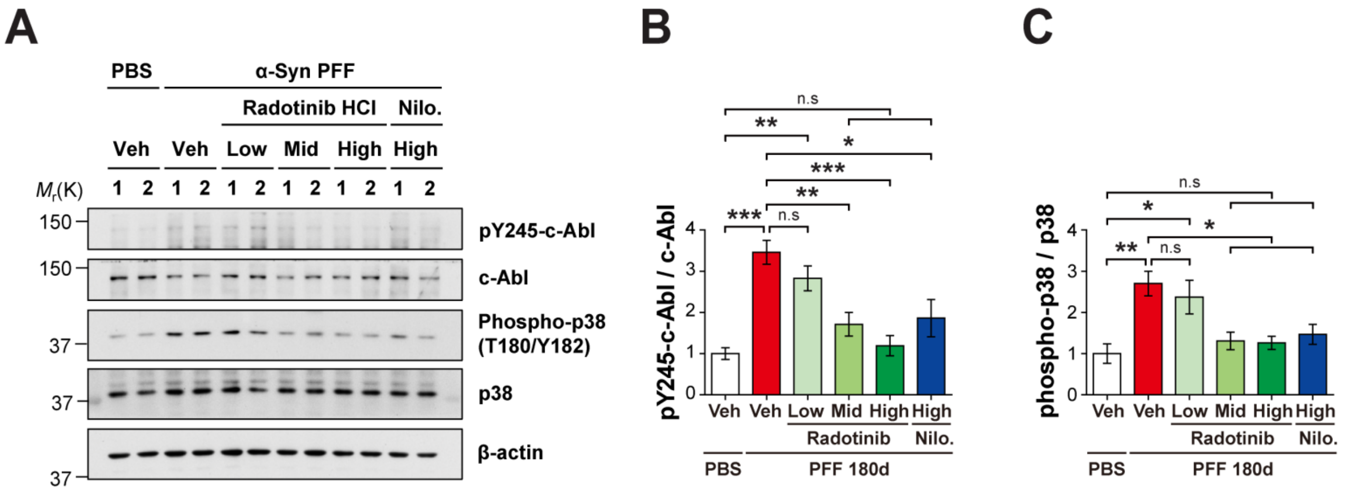

Figure S1. Radotinib HCl reduces α-synuclein PFF-induced c-Abl activation and p38 MAPK phosphorylation *in vivo*. (A) Representative immunoblots with ventral midbrain tissue lystates in PBS and α-synuclein PFF stereotaxic injected mice treated with vehicle or Radotinib HCl and Nilotinib. (B) Quantification of the levels of pY245-c-Abl normalized to c-Abl. (C) Quantification of the levels of phospho-p38 MAPK normalized to p38 MAPK. Bars indicate mean ± S.E.M. (n=4). Statistical significance was determined by using one-way ANOVA with Tukey's correction; $^*P < 0.05$, $^{**}P < 0.01$, $^{***}P < 0.001$. n.s., not significant. Radotinib, Radotinib HCl. Nilo., Nilotinib.
